# The Precarious In-Between: Early Retirement for Those Neither Old Nor Young Enough, Rich Nor Poor Enough

**DOI:** 10.1093/geroni/igaf122.122

**Published:** 2025-12-31

**Authors:** Crystal Kwan

**Affiliations:** The Hong Kong Polytechnic University, Hong Kong, Hong Kong, China (People’s Republic)

## Abstract

**Research Aim:**

This study explores the perspectives, lived experiences, and expectations of early retirees with lower incomes as they adjust to retirement in Hong Kong.

**Methods:**

We conducted a qualitative study with 50 early retirees (before age 65) classified as lower-income per Hong Kong’s Low Income Working Family Allowance (LIFA) criteria. Over ten weeks, data was collected through semi-structured interviews lasting 45-75 minutes and supplemented by weekly photo-diary entries. A resource-based dynamic perspective was the theoretical framework, guiding the coding. Thematic analysis of both visual and textual data helped extract themes.

**Findings:**

Most participants faced involuntary retirement due to economic recession, ageism, work pressure, caregiving demands, and health issues. A major theme was the ironic transition from long work hours to sudden free time, leading to either relief and pursuit of interests or feelings of aimlessness. Financial concerns were prevalent; however, many adapted by embracing a simpler lifestyle. Ambivalence about the adequacy of their limited savings for retirement was a common sentiment. Another significant theme was the positive impact of developing healthy habits and the additional challenges faced by subgroups dealing with caregiving and housing issues.

**Conclusions and Implications:**

This study underscores the necessity for targeted policies and early intervention programs to assist this overlooked group at risk of poverty, proposing strategies to enhance their agency and overcome ambivalence to facilitate a positive adjustment to retirement.

